# A Novel Method for Extraction of Galegine by Molecularly Imprinted Polymer (MIP) Technique Reinforced with Graphene Oxide and Its Evaluation Using Polarography

**DOI:** 10.1155/2020/3646712

**Published:** 2020-03-01

**Authors:** M. Azimi, M. Ahmadi Golsefidi, A. Varasteh Moradi, M. Ebadii, R. Zafar Mehrabian

**Affiliations:** Department of Chemistry, Faculty of Sciences, Gorgan Branch, Islamic Azad University, Gorgan, Iran

## Abstract

*Galega officinalis* products have been used for the control of diabetes (type 2) across the world. Experimental and clinical evaluations of galegine substance produced by a medicinal plant (*Galega officinalis*) provided the pharmacological and chemical basis for metformin discovery which was confirmed for diabetes therapy. In this paper, the molecularly imprinted polymer (MIP) was synthesized for galegine, using galegine as a template molecule, methacrylic acid (MAA) as a functional monomer, ethylene glycol dimethacrylate (EGDMA) as a cross-linker, azobisisobutyronitrile (AIBN) as a reaction initiator, and acetonitrile as a solvent. The assisted functional groups, morphology, topographic image of surface, and crystalline structure of synthesized MIP were characterized by FT-IR spectroscopy, field emission scanning electron microscopy (FE-SEM), atomic force microscopy (AFM) images, and XRD diffraction pattern techniques, respectively. Also, the performance of the mentioned electrode was quantified and qualified by the differential pulse voltammetry technique (DPV). The galegine amount was determined with the polarographic technique. In this research, the galegine extraction conditions were optimized and graphene nanoparticles were used to increase the adsorption. In addition, different parameters affecting extraction were investigated such as MIP adsorbent amount, pH of solution, effect of the surfactant, and ionic compound to achieve high recovery percent. The recovery percent, limit of detection (LOD), limit of quantification (LOQ), and relative standard deviation (RSD %) were 4.101 *μ*g·mL^−1^, 12.427 *μ*g·mL^−1^, and 1.199% (*n* = 3), respectively. The results show that the prepared MIP can be used as an effective and inexpensive adsorbent for preconcentration and galegine extraction from a natural sample. It is noteworthy that this developed method was used successfully to determine galegine extracted from *Galega officinalis L*.

## 1. Introduction

**Figure 1:**
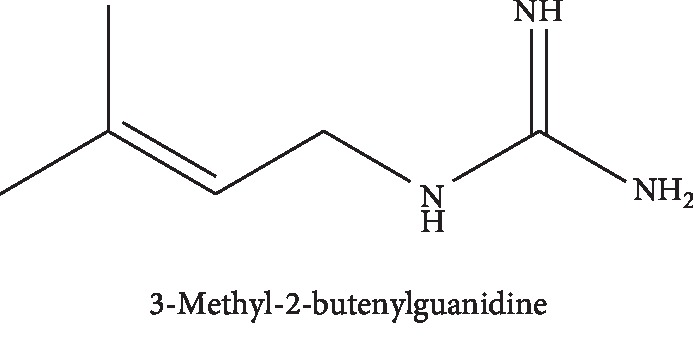
Galegine chemical structure.

Today, in many countries, the use of medicinal herbs has been of great interest for the treatment of diseases [1]. One of these plants is *Galega officinalis L*., commonly referred to as goat's rue, a native plant of Southern Europe and Western Asia, which is grown from the Fabaceae family and well-grown in humid regions at about 25°C [2]. *G. officinalis L*. has been used as an antidiabetes drug [3, 4]. Further studies showed that the *Galega officinalis* extract has an effect on human platelet aggregation and body weight loss [5]. The effective substance of *Galega* is galegine, with the formula of C_6_H_13_N_3_, which was first recognized by Tanret and isolated from this plant [6]. Galegine is a guanidine derivative that (3-methyl-2-butenylguanidine) was known by Perkop and Spath (Figure 1).

Other compounds derived from *Galega* include flavonoids, glycosides, alkaloids, 4-hydroxygalgens, tannins, saponins, sucrose, and fatty oils [7]. Galegine reduces blood sugar [8] and has an anticoagulant effect [9], decreases blood pressure [10], and increases the production of milk in sheep [11]. Due to the importance of galegine and its small amount in the plant, a method of dissecting should be used in order to extract with the high purity that its sensitivity, repeatability, and the measurement accuracy should be high. So far, several methods have been investigated for extracting galegine using various techniques by researchers [12, 13]. For example, solvent extraction [14, 15] and supercritical fluid extraction [16] are among these methods. Measurement and extraction of galegine in the natural sample are difficult due to factors such as a low concentration of this material in the sample and matrix effects. To overcome this problem, numerous methods have been developed such as preconcentration and using more sensitive instruments. The precondensation has been developed by solid phase extraction (SPE), liquid-liquid, precipitation, and coprecipitation. Among the aforementioned methods, the solid-phase extraction was more valuable due to the simple issue, low consumption of organic solvent and factors in high concentration [17]. In this method, a solid phase and a liquid phase are used to separate the chemical or natural compounds in the solution. The superiority of the solid-phase extraction method is its selectivity [18]. In this method, the desired effective material is extracted from the liquid phase into the solid phase. Recently, the method of molecularly imprinted polymer (MIP) has been introduced as one of the most powerful techniques for the extraction and separation of the natural compounds [19]. This technique is used for the preparation, extraction, and preconcentration of drugs, natural compounds, and contaminants available in the environment, water, biological materials, and food products [20]. In the extraction method with MIP between functional groups of the target molecule and functional groups of the monomer, an intermolecular reaction is generated and diagnostic sites are obtained. These sites are tied up by polymerization of functional monomers with a high concentration of a transverse connector. Then, they are removed by molecule solvent washing the template. The obtained polymer is called molecularly imprinted polymer (MIP) [21]. This technique has been used by Gama [22] and potentially used in extraction of many samples, such as the separation of chiral molecule [23], biosensors [24], and isolation of biochemical compounds, antibody simulation, and simulation of enzyme decomposition [25]. Molecular imprinting polymers have features such as low cost and easy synthesis, the high stability to physical and chemical conditions, reusability, fast polymer adsorption of templates, and potential application to a wide range of target molecules [26, 27]. The aim of this study was to provide a solid extraction phase based on molecular imprinting polymer for extraction of galegine from a herbal medicine, *G. officinalis L*., and the created cities which took stronger once the reaction of functional groups and high concentration cross-linkers happened due to the polymerization process. The effective factors on MIP were mass optimized, and then it was used to extract natural samples. This study was performed for the first time by the electrochemical method to evaluate the efficiency of the method. The electrochemical method has a special place as fast, simple, and low-cost systems with high selectivity and repetitive effects. The use of the electrochemical methods in the recent decades has been widely used for the quantitative determination of various organic and mineral species through preconcentration of the samples. For this purpose, the polarography instrument was used for measuring galegine.

## 2. Experimental

### 2.1. Materials, Methods, and Instruments

Galegine sulfate was purchased from Select Lab. Co., with a purity of 98%. *Galega officinalis L*. was purchased from Zardband Company, which produces raw materials for pharmaceutical companies, methacrylic acid (MAA), azobisisobutyronitrile (AIBN), ethylene glycol dimethacrylate (EGDMA), acetonitrile, graphene oxide, and other solvents and chemical compositions were purchased with a purity of 98% from Sigma-Aldrich. All electrochemical measurements were selected using the VA Computrace 797 Polarographic instrument manufactured by Metrohm, Switzerland. The roughness of the synthesized MIP was studied by the atomic force microscopy (AFM, DME-95-50E, German). The following instruments were used to investigate the internal structure and polymer porosity as follows: the XRD instrument (PHILIPS Company, Netherlands), the FT-IR instrument (Thermo Company AVATAR, USA), and the FE-SEM instrument (TESCAN MIRA3 model, Czech Republic).

### 2.2. Synthesis of Molecularly Imprinted Polymer (MIP)

Methacrylic acid was used as a monomer [28], and EGDMA was used as a short, strong, and flexible bonding bridge between methacrylate groups [29]. In order to initiate the polymerization process in MIP synthesis, it is necessary to use a primer material.

**Figure 2:**
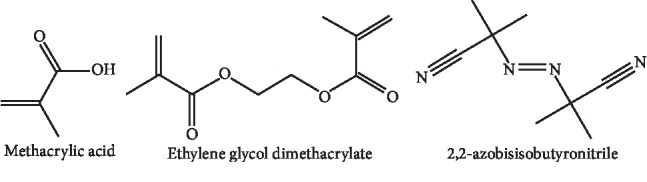
Structure of the raw materials of the reaction.

Thermal decomposition of the primers is the most common source of free radicals in the process of MIP formation. In this regard, the azobisisobutyronitrile compound (ALBN) was used. Since acetonitrile is an organic porogenic solvent, it was used as the solvent [30]. For the synthesis of molecularly imprinted polymers, the following materials were used: 1 mmol of the target molecule (galegine), 0.5 ml of the monomer of methacrylic acid (MAA, 98%), 5.6 ml of ethylene glycol methyl acrylate (EGDMA) as a cross-linker, and 0.05 gr of 2,2- azobisisobutyronitrile (AIBN) reaction initiator [31]. The broad structure of the main raw material of the reaction is given in Figure 2.

**Figure 3:**
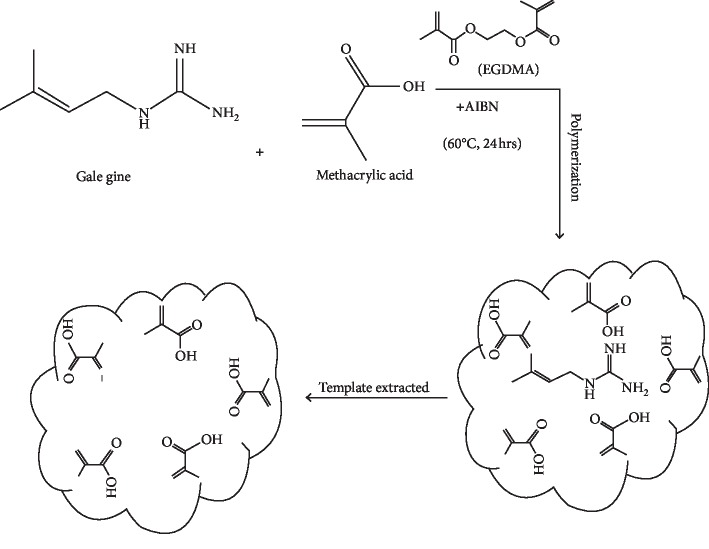
A schematic of trapped galegine molecule in the synthesized MIP.

All materials were dissolved by acetonitrile and then were oxygenated for 20 minutes and were placed in an ultrasonic bath to make the mixture homogeneous. Polymerization reaction started in mass and with the radical mechanism. The reaction has been completed for 24 hours in the bain-marie bath at a temperature of 70°C. The polymer obtained after wrapping and softening in the masonry was screened and homogenized from wire mesh sieves to mesh sizes ranging from 1000 to 2000 micrometers (Figure 3).

The molecularly imprinted polymers were washed with solvents (methanol (twice) and water (once)) to remove the molecule of the template (galegine). The ratio of methanol and water to MIP was 1 : 10 for 1 hour (each step). Then, the solid phase was dried at 70°C. To prepare NIP, all steps of MIP synthesis were performed without the addition of the template galegine. After the formation of the polymer, it is not necessary to remove the target molecule.

### 2.3. Preparation of Standard Solutions and Real Samples

The stock of standard solution (galegine) was prepared at 100 mg·l^−1^ in water. Aqueous solutions were prepared with concentrations of 5, 10, 15, 20, and 25 mg·l^−1^. In order to determine the MIP penetration coefficient, it is necessary to load some molecularly imprinted polymer in the presence of target molecules and to carry out the remaining amount of the polarographic test. To extract the galegine, 0.02 gr of the synthesized MIP was added to the standard solutions. Then, they were placed in an ultrasonic bath for 20 minutes and it was filtered.

### 2.4. Polarographic Device Conditions

In this study, all quantitative and qualitative electrochemical measurements were performed by the polarographic device (797 Metrohm) with a three-electrode system, which contained the droplet mercury electrode (DME), saturated calomel electrode (SCE), and Pt rod electrode used as working, reference, and counter electrodes, respectively. The instrument was set as given in Table 1.

The detector responses were proportionated to the obtained peak area which led to quantifying the extraction rate of the sample. The amount of sample was determined from the comparison of the peak area of the sample with the peak area of the standard solution for the diluted (0.1 ml of the standard galegine sulfate to 20 ml) solution. Before polarography testing, the prepared solution was purged with N_2_ gas for 5 min at room temperature.

### 2.5. Calculation and Evaluation of the Penetration Coefficient of the Polymer

The penetration coefficient of the polymer (*Q*) was calculated by equation (1), where there is a polymer penetration coefficient that states the amounts of penetrated molecules (mg, *μ*g, or mM) in 1 g of the polymer. From the following equation, *C*_e_ is the concentration after loading, *C*_o_ is the initial concentration of the solution in mg·l^−1^, *V* is the volume of solution in liters, and *m* is the polymer mass; the penetration coefficient is calculated as follows:

(1) $$\begin{array}{c} \;Q=\frac{C_{\text{o}}-C_{\text{e}}}{m}\times V. \end{array}$$

## 3. Results and Discussion

### 3.1. XRD Study

**Figure 4:**
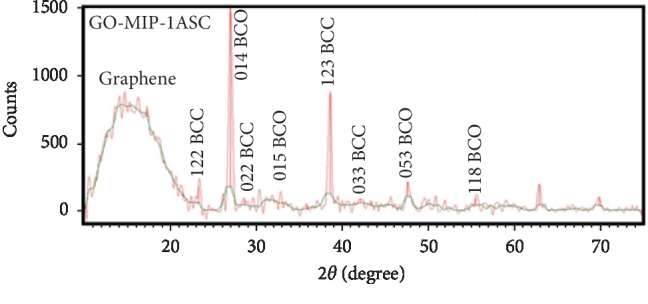
XRD spectrum for the GO-MIP molecular-imprinted nanopolymer.

The X-ray diffraction test was used to study the crystallography of the particles to estimate the size of the crystals and to analyze how they were involved in the GO-MIP nanocomposite structure. Noteworthy, as far as our knowledge, this polymer (MIP) was built for the first time and there were no reports on its structure. The obtained results of XRD have shown three crystal structures such as simple cubic, orthorhombic, and hexagonal cubes in the polymerized MIP. The dimensions of the cubic dimensions were *a* = *b* = *c* = 8.835 A while *α* = *β* = γ = 90°, and also the dimensions and angles of orthorhombic were *a*(50.821) ≠ *b*(10.353) ≠ *c*(13.99)A. Studies show that two mentioned crystallographic structures were related to the MIP backbone; referencing to the previous studies, the dimensions and angles of hexagonal were *a*(2.456) = *b*(2.456) ≠ *c*(16.74)A and *α*(90°) = *β*(90°) ≠ *γ*(120°) that was related to the structure of graphene oxide mixed with MIP [32]. The related Miller's indexes (hkl) for synthesized crystals were given as follows: the broad peak at 2*θ* = 20 was related to graphene oxide. Peaks with plans: 122, 022, 123, and 033 appeared at 2*θ* = 24.66, 28.58, 38.58, and 43.2 for the (body-centered cubic) BCC crystal, respectively. Also, the plans: 014, 015, 133, and 118 were found at 27.00, 33.00, 35.80, and 48.01 relating to the crystal (base-centered orthorhombic) BCO, respectively (Figure 4).

### 3.2. FE-SEM Studies

**Figure 5:**
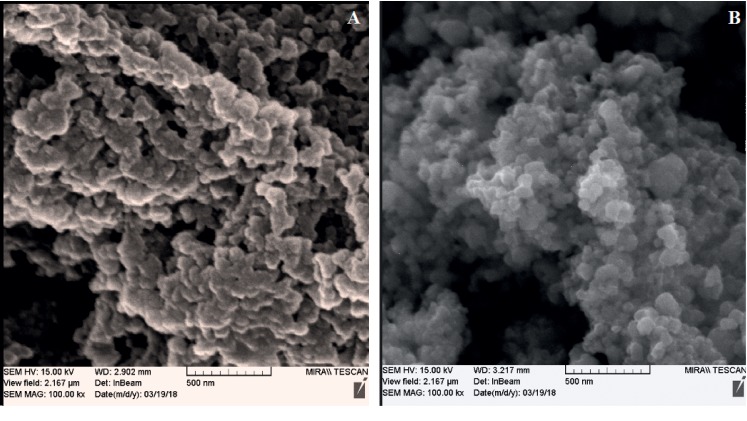
FE-SEM images with the magnification of 500: (a) molecularly imprinted polymer with graphene oxide and (b) molecular-imprinted polymer.

The FE-SEM was used to capture the molecularly imprinted polymer. In Figure 5, the FE-SEM images of MIP and GO-MIP are shown and it was found that the particle size were in the range of 100–300 nm. As can be seen, the spherical structure morphology was well formed for the GO-MIP mixture nanocomposite, whereas the MIP has shown smaller cavities and agglomeration was carried out. It seems that galegine molecules were located in the polymer template once the graphene nanolayers were mixed with GO-MIP.

### 3.3. FT-IR Investigation

**Figure 6:**
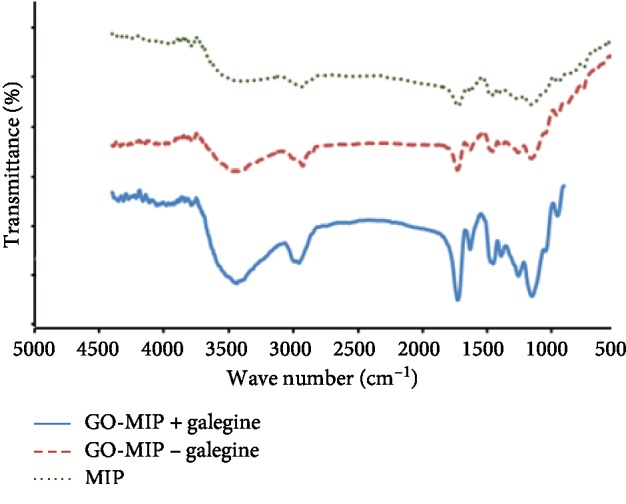
Recorded IR spectra of polymers.

The associated functional groups were investigated using the FT-IR technique for the synthesized MIP, GO-MIP, and GO-MIP including galegine.  Figure 6 shows that the observed peak at 3435 cm^−1^ was attributed to OH groups of methacrylic acid [33]. The peak available about 1730 cm^−1^ was related to the C=O bond, and the vibrations related to the C-O bonds were displayed at 1150 cm^−1^. Therefore, it could be concluded that the polymerization process was well performed due to the reaction between methacrylic acid and EGDMA. On the other hand, both the FT-IR spectra of MIP and GO-MIP were very similar to each other, and it led to that the graphene oxide peaks were superimposed with the appeared peaks of MIP.

### 3.4. AFM Analysis

**Figure 7:**
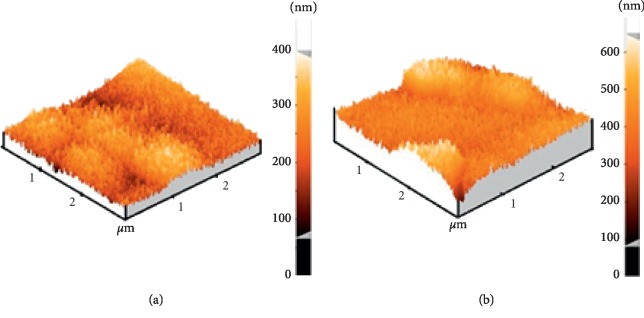
AFM images: (a) molecular-imprinted polymer and (b) graphene-reinforced molecularly imprinted polymer.

AFM was used to obtain qualitative information (topography). The surface (3 × 3 *μ*m) of GO-MIP and MIP was scanned (Figure 7), and the roughness of both sample surfaces was displayed.

Noteworthy, it was found that the particle size was 44.8 nm and 56.7 nm, for the synthesized MIP and GO-MIP samples, respectively. It could be concluded that roughness was increased due to the graphene oxide in the imprinted composite. It can be suggested that the chain of the polymer was entrapped in the presence of GO, which led it to bigger cavities. On the other hand, the adsorbance properties of GO carried agglomerations.

### 3.5. Weight of MIP as Acceptor Phase

**Figure 8:**
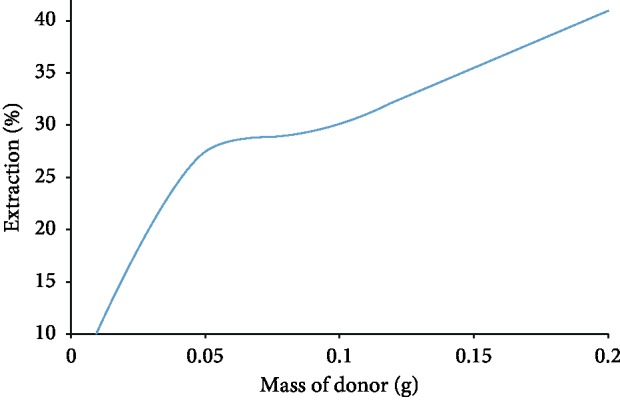
The effect of adsorbent mass changes on the galegine extraction rate.

Different amounts of the synthesized MIP (0.01, 0.02, 0.05, 0.08, 0.1, 0.2, 0.3, and 0.5 g) were ultrasound in 10 ml of galegine sulfate solution (10 mg·l^−1^) for 20 minutes. Then, the mixture was filtered and was prepared to measure the adsorption with the polarographic technique.  Figure 8 shows that how the adsorbent amount has been affected by results. It was found that the amount of the extracted galegine was increased with increasing of the adsorbent. It was carried out due to increasing the adsorbent surface with more available sites. Therefore, the value of 0.05 g was optimal as displayed in Figure 8; however, the mentioned amount was economically reasonable.

### 3.6. Amount of Graphene Oxide (GO) in MIP

**Figure 9:**
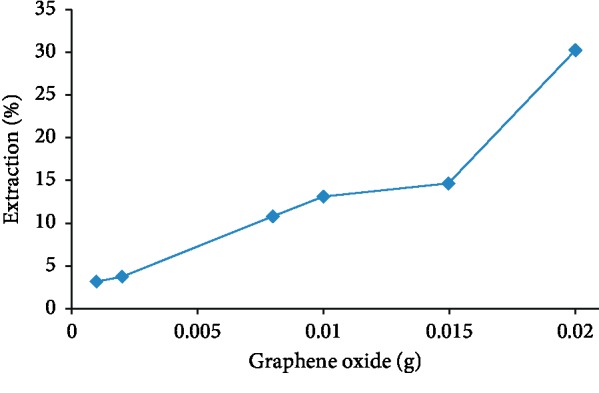
The effect of graphene content on galegine adsorption.

Graphene oxide was used as an adsorbent layer to increase the MIP uptake. Graphene oxide has a two-dimensional carbon structure similar to a honeycomb grid and a thickness of an atomic layer that one of the extraordinary properties of graphene oxide was its high surface [34]. Graphene oxide with amounts (0.001, 0.002, 0.008, 0.01, 0.015, and 0.02 g) was added to GO-MIP to achieve a high surface area. The optimum amount of the adsorbent (0.05 g GO-MIP) was ultrasonically mixed with the galegine solution (10 mg·l^−1^) for 20 minutes. The adsorbed galegine by GO-MIP was filtered, and the rest galegine in the solution was measured by polarography. The adsorbed galegine was increased once the GO was increased in the synthesized MIP. It was found that the highest amount of the adsorption was achieved with 0.02 g graphene oxide (Figure 9).

### 3.7. Extraction Time

**Figure 10:**
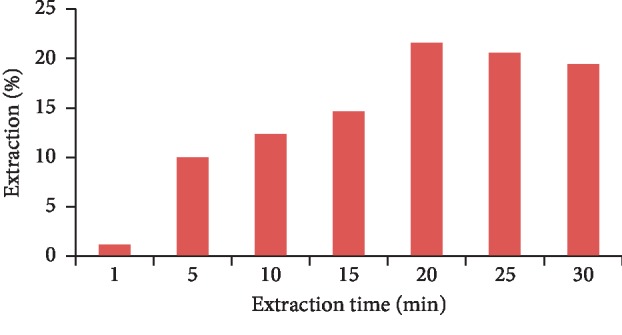
Diagram of the galegine adsorption (through the extraction %) from aqueous solution using GO-MIP in the different time.

To get the optimum adsorption time, the solution containing the analyte (10 mg·l^−1^) was in contact with MIP on a variety of times (1, 5, 10, 15, 20, 25, and 30 minutes). Then, the rest galegine was measured. The survey results of the contact time on the galegine adsorption have shown that, however, the adsorption galegine was increased with enriched MIP with the GO for the initial times, but also it was diminished with increasing time from the galegine solution (10 mg·l^−1^).  Figure 10 shows that the adsorption rate was decreased while the extraction time was passed from 20 minutes. It could be described that the activated sites were blocked due to the adsorbed galegine on the adsorbent surface in first 20 minutes. Thus, the optimum time to capture galegine by MIP was 20 minutes in this study (Figure 10).

### 3.8. pH of Liquid Phase

**Figure 11:**
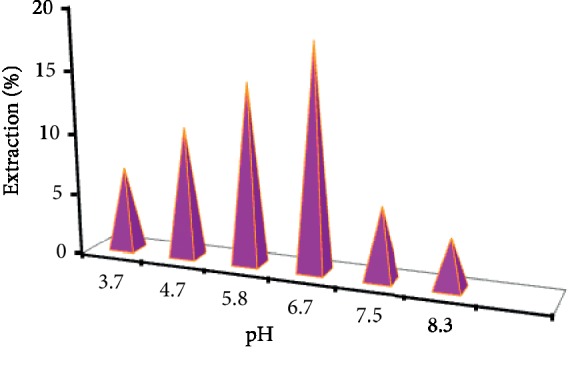
Diagram of the galegine adsorption (through the extraction %) from aqueous solution using GO-MIP in different pH.

The influence of pH of the liquid phase was investigated to determine the adsorption rate and capacity from the standard solution of galegine 10 mg·l^−1^. The pH of the solution was adjusted by diluted HCl as 3.5, 4.5, 5.5, 6.5, 7.5, and 8.5. The process of this section was similar to the last part as the amount of 0.05 g of GO-MIP was ultrasonically mixed in the solution containing the analyte for 20 minutes. This study has shown that the pH significantly affected the galegine adsorption by the mentioned adsorbent. The extracted galegine curve (Figure 11) has displayed that the adsorption was linearly increased with increasing pH from 3.5 to 6.5 and decreased once it was increased from 6.5. The mid of linear curve (pH = 5.0) was used for the extraction process. Noteworthy, it could be described as follows: the pK_a, galegine_ = 11.96 decreased the pKa galegine adsorption rate at pH less than 4.5 and higher than 6.5, and it appears that the pH has an effect on the charge loading of the adsorbent surface and the analyte particles. Therefore, pH changes will affect the adsorption process and the proton can react with active factor groups on the surface of the adsorbent or analyte molecules. As a result, the protonated galegine was electrically polarized and then they would be appositively oriented to the adsorbent surface while pH was adjusted in the range 3.5–6.5 and the adsorption was subsequently increased as shown in Figure 11. However, the founding results were confirmed by Ansari and Ghorbani [34].

### 3.9. Influence of Surfactant

**Figure 12:**
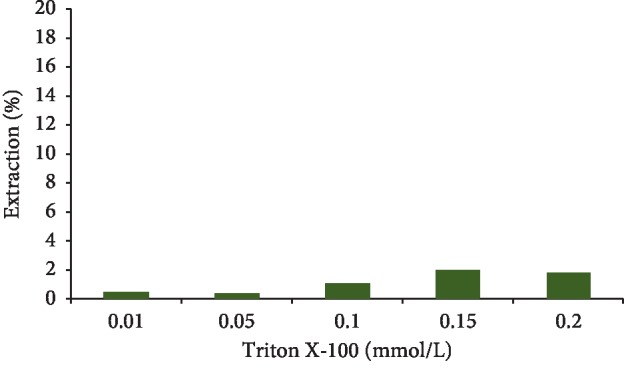
The effect of surfactant on the galegine extraction rate.

Triton X-100 is a commercial nonionic detergent that has a hydrophilic oxide polyethylene chain and a hydrophobic hydrocarbon group. The chemical name of Triton X-100 is ethoxylate octyphenol that is viscous at room temperature and has a density of 1.07 g m·l^−1^. Biodegradability in a wide range of temperatures is among the benefits of this detergent [36]. For this purpose, the values of Triton X-100 (0.01, 0.05, 0.1, 0.15, and 0.2 mmol·L^−1^) were used. It is expected that the surface tension is reduced by increasing the surfactant and subsequently, the adsorption rate will reduce. It resulted that the solubility of galegine was reduced in water due to the decreasing surface tension between galegine and GO-MIP once the tiny amount of Triton X-100 was added. It led to reduce the galegine adsorption on the GO-MIP (Figure 12).

### 3.10. Effect of Ion Strength

Theoretically, we have expected that by increasing the ionic composition such as NaCl or KCl to the solution, it has practically resulted that the extraction of galegine was not changed by increasing a variety of amount of NaCl (0.01, 0.05, 0.1, 0.2, 0.5, and 1 W/V%) in the following condition: galegine solution at 10 mg·l^−1^, pH = 5, *t* = 20 min, and 0.05 g GO-MIP.

### 3.11. Investigation of Adsorption Isotherms

Adsorption properties have been usually described by adsorption equilibrium isotherms. In this study, the MIP-based galegine equilibrium adsorption was performed by examining the adsorption isotherm by selecting the basic conditions including preparation of solutions at concentrations of 5, 10, 20, 30, 50 mg·l^−1^, and 0.05 g MIP and pH = 5. Both Langmuir and Freundlich isotherms were used to study the experimental results. The linear form of the Langmuir model was used to investigate the adsorption phenomena as follows [37]:

(2) $$\begin{array}{c} \frac{1}{q_{\text{e}}}=\frac{1}{\left(q_{\max}\cdot K_{\text{L}}\cdot C_{\text{e}}\right)}+\frac{1}{q_{\max}}, \end{array}$$

where *q*_e_ is the maximum amount of the adsorbed galegine per unit mass of the adsorbent (mg·g^−1^), *C*_e_ is the concentration of the galegine solution (mg·l^−1^), and *K*_L_ is the equilibrium constant of the adsorbance (l·mg^−1^). In the Freundlich model, by plotting the logarithmic curve *q*_e_, we can calculate the values of *n* and *K*_L_ from intercept and slop, respectively. *K*_L_ is the expression of the adsorbed galegine amount per unit of equilibrium concentration and *n* represents the distribution of the adsorbed material on the adsorbent surface 1/*n* with values between 0-1 that indicates the heterogeneity of the surface; the heterogeneity of the surface is increased as *n* is approached zero:

(3) $$\begin{array}{c} \ln\;q_{\text{e}}=\ln\;K_{\text{L}}+\frac{1}{n}\cdot \;\ln\;q_{\max}. \end{array}$$

**Figure 13:**
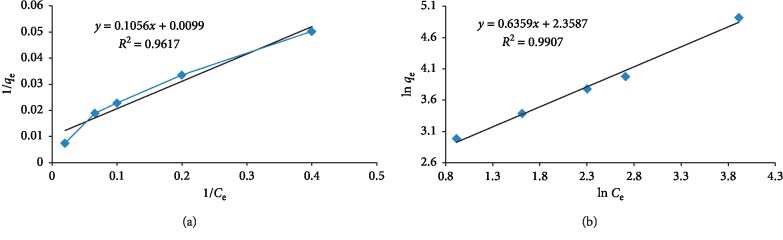
(a) Nanosensitive Langmuir adsorption isotherms; (b) nanosensitive Freundlich adsorption isotherms in the galegine adsorption (adsorbent dosage 0.05 g, contact time 20 min, and optimal pH = 5).

Different models of the adsorption isotherm were analyzed by comparing the coefficient of determination (*R*^2^). It was found that the results were entirely fitted with *R*^2^ through the Langmuir compared to Freundlich isotherm for the galegine adsorption process by MIP (*R*^2^ = 0.99) (Figure 13).

The values of *K*_L_ and *q*_m_ (maximum adsorption capacity) were calculated. Its values are presented in Table 2. According to these results, it can be said that the adsorption sites on the synthesized GO-MIP were normally distributed on the surface of the polymeric molecular template. The values of *K*_L_ and *n* are given in Table 2.

In overall, these results showed that there was an appropriate interaction between galegine molecules and GO-MIP levels. Using the obtained data, the process was optimized as the polymer content of the molecular imprinted 0.05 g, the amount of graphene oxide 0.02 g, pH = 5, and the contact time 20 minutes. The solid phase as a molecular imprinted was a useful technique to the extraction of galegine. To the best of our knowledge, it was not reported previously.

### 3.12. Extraction of Galegine from Natural Samples Using GO-MIP

In order to extract galegine from the *Galega* plant, 0.05 g of the prepared solid phase was mixed with 10 ml of the hydroalcoholic galegine extracted in the ultrasonic bath for 20 minutes. The solution was filtered, and the subfiltration solution was analyzed by a polarographic technique.

### 3.13. Validation of the Method

The analysis features of the proposed method were studied. After optimizing the affecting factors on the galegine extraction by the MIP technique, the calibration curve was linearized in the optimal concentration range (1–100 *μ*g·ml^−1^) and the assistance equation was as *y* = 0.9648*x* + 18.573, while the correlation coefficient (*R*^2^) was 0.9985, as well. The limit of detection (LOD), limit of quantification (LOQ), and relative standard deviation (RSD%) was calculated 4.101 *μ*g·ml^−1^, 12.427 *μ*g·ml^−1^, and 1.199%, respectively.

### 3.14. Comparison of Previous Studies with the Proposed Method

The results of the present study were compared with previous research studies' findings that are displayed in Table 3 for galegine process. It was found that the extraction of galegine by GO-MIP was significantly efficient compared to other studies.

## 4. Conclusions

In this study, galegine extraction from the *Galega* leaves was selected by the MIP as the main subject of the research. The adsorbent was synthesized based on methacrylic acid (MAA). It was characteristics by XRD, FE-SEM, AFM, and FT-IR. The fabricated GO-MIP as a selective and sensitive solid phase adsorbent was used to extract galegine through the preconcentration condition such as easy preparation, selectivity and high sensitivity, good repeatability, wide linear domain, and low limit of detection (LOD = 0.157 *μ*g·mL^−1^; LOQ = 0.157 *μ*g·mL^−1^) and has been successfully used to determine the galegine drug in the plant sample. In addition to the high molecular bias in polymer bonding sites, these sites also play a vital role in achieving the best adsorption of the analyte and successful extraction of molecularly imprinted polymers to appropriate selection of polymer size, molecular shape, nanoparticle size, ambient pH, and contact time.

## Figures and Tables

**Table 1:** Metrological parameters of the polarography device.

Work conditions	Parameters and amounts
Working electrode	DME
Stirrer speed	2000
Mode	DP
Purge time	300 s
Equilibration time	5 s
Pulse amplitude	50 mv
Start potential	100 mv
End potential	−350 mv
Voltage step	6 mv
Voltage step time	0.65
Sweep rate	10 mv/s
Peak potential	−170 mv

**Table 2:** Parameters of Langmuir and Freundlich isotherms on galegine adsorption on the GO-MIP surface.

Adsorption isotherms	Langmuir isotherm			Freundlich isotherm		
Parameters	*R* ^2^	*K*	*q*_max_ (mg/g)	*R* ^2^	*n*	*K*_L_
	0.8412	7.50	15.091	0.9862	1.72	218.0218

**Table 3:** Comparison of the results of previous studies to determination of galegine with the result of the proposed method.

Date	Concentration range	Reference	Method
2016	mg/g 3.412	Davoodi [16] et al	Soxhlet
2016	mg/g 3.3932	Davoodi [16] et al	Supercritical fluid extraction
This study	12.4 *μ*g/g		Polarography with MIP technique

## Data Availability

The data used to support the findings of this study are available from the corresponding author upon request.
