# Erratum to “A Novel Method for Extraction of Galegine by Molecularly Imprinted Polymer (MIP) Technique Reinforced with Graphene Oxide and Its Evaluation Using Polarography”

**DOI:** 10.1155/2021/3548023

**Published:** 2021-02-10

**Authors:** M. Azimi, M. Ahmadi Golsefidi, A. Varasteh Moradi, M. Ebadi, R. Zafar Mehrabian

**Affiliations:** Department of Chemistry, Faculty of Sciences, Gorgan Branch, Islamic Azad University, Gorgan, Iran

In the article titled “A Novel Method for Extraction of Galegine by Molecularly Imprinted Polymer (MIP) Technique Reinforced with Graphene Oxide and Its Evaluation Using Polarography” [1], there was a spell error in author M. Ebadii's name in the author list, where “M. Ebadii” should have read as “M. Ebadi.” This is corrected as shown above.

The error was introduced during the production process of the article, and Hindawi apologises for causing this error in the article.
